# The impact of respite care from the perspectives and experiences of people with amyotrophic lateral sclerosis and their care partners: a qualitative study

**DOI:** 10.1186/s12904-022-00919-2

**Published:** 2022-02-28

**Authors:** Julia M. Wu, Mallorie T. Tam, Kirsten Buch, Fouziah Khairati, Laurissa Wilson, Elizabeth Bannerman, Alexandra Guerrero, Andrew Eisen, Wendy Toyer, Travis Stevenson, Julie M. Robillard

**Affiliations:** 1 Department of Medicine, Division of Neurology, The University of British Columbia, Vancouver, BC Canada; 2 BC Children’s & Women’s Hospital, 4480 Oak Street, Room B402 Shaughnessy, Vancouver, BC V6H 3N1 Canada; 3 ALS Society of BC, Vancouver, BC Canada

**Keywords:** Amyotrophic lateral sclerosis, Respite care, Qualitative research, Caregiving

## Abstract

**Background:**

Respite care provides caregiving support to people with amyotrophic lateral sclerosis (ALS) and their care partners by providing the care partner with temporary relief from their caregiving duties. The aim of this study was to explore the impact of respite care through the perspectives and lived experiences of people with ALS and their care partners.

**Methods:**

Thirty-one dyads (62 participants) of people with ALS and their care partners were assigned to either the control group or the respite care intervention. Respite care was provided in the form of home-based services. Semi-structured interviews were conducted with participants at baseline and after a six-month period to gather perspectives on ALS caregiving, perceptions of respite care, and the respite care experience. Interviews were transcribed and subjected to thematic analysis.

**Results:**

Caregiving challenges specific to the care partner and the patient-care partnership relationship were identified. Overall, people with ALS and care partners responded positively to in-home respite care and reported improved relationship quality, more time for the care partner to pursue personal commitments or take a break, and improved emotional well-being for both the person with ALS and the care partner. Barriers and concerns were raised surrounding privacy and staff consistency.

**Conclusion:**

This study highlights respite care as a critical tool to alleviate caregiving challenges and support the needs of people with ALS and their care partners. Engagement with the ALS community and formal evaluations of respite care services should be prioritized in order to minimize barriers and best meet the needs of people with ALS and their care partners.

## Background

Amyotrophic lateral sclerosis (ALS) is a progressive, neurodegenerative disease that impacts the physical, communication, and cognitive functioning of those affected [1, 2]. People with ALS experience progressive paralysis of all voluntary muscles and can suffer from loss of ambulation, dysarthria, and dysphagia [3]. With no cure or treatment available, the majority of people diagnosed with ALS die within three to five years of symptom onset [3].

Spouses or partners frequently take on a central role as an informal caregiver to provide emotional and physical support for their loved ones. Receiving care from a partner allows the person with ALS to live at home in a familiar environment with their family, often promoting their quality of life [4]. However, given the sudden onset and rapid deterioration of the disease, care partners are faced with increasing demands and responsibilities, high levels of dependency by their partner, and a shifting nature of the patient-care partner relationship [2, 5]. Many care partners maintain employment and face additional responsibilities to caregiving such as work and family [1], making the caregiver role more difficult to manage. As a result of these combined difficulties and the close intertwinement of life with that of the patient’s, care partners are at risk of increased burden, psychological distress, and impaired quality of life [3, 6–8].

One way to alleviate the stress and burnout experienced by ALS care partners is through the provision of respite care. According to the ALS Association (2022), respite care can be defined as an interval of rest or relief that provides opportunity for the family caregiver to take a break from the daily care of their loved one. Respite care can be offered as home-based services which typically involves a trained professional coming into the home to provide necessary care during a period of time when the family caregiver is away [9]. Other forms of respite care can include allowing the care recipient to receive the care they need at a facility or residence on a short-term basis [9]. A period of respite can vary in time from hours to days, depending on what is decided between the care recipient and their caregiver [9]. Previous research has shown respite care to be an effective source of support for care partners and individuals with neurodegenerative diseases [6, 10, 11]. Studies exploring respite care have reported benefits for the care partner including decreased caregiver burden, more time to take care of themselves and other home responsibilities, and a greater level of self-compassion when evaluating their caregiver performance [6, 11]. Respite care has also been shown to benefit the care recipient by reducing behavioural problems and improving sleep quality [6, 10].

Despite the potential benefits, respite care among the ALS community remains underused and access to the service is limited [6, 12]. To date, there is a lack of evidence surrounding the effectiveness of respite care in supporting the needs of families with ALS. To address this gap in the literature, the aim of this study was to explore the impact of respite care through the perspectives and lived experiences of people living with ALS and their care partners.

## Methods

### Design

This study adopted a qualitative description design for its aim to uncover the rich narratives and lived experiences of individuals experiencing a phenomena of interest [13, 14]. Semi-structured interviews were conducted to explore the perspectives and lived experiences surrounding respite care among people with ALS and their care partners. For this study, respite care was provided in the form of home-based services. Aligned with the philosophies and principles of healthcare research, qualitative description serves as a valuable tool in promoting patient-centred care as it provides a vehicle for individuals to share and find meaning in their lived experiences [15]. To better contextualize the impact of respite care, we supplemented our qualitative design with standardised quantitative assessments to measure physical, cognitive, and psychological measures of people with ALS and their care partners.

### Participants

Thirty-three dyads (66 participants) of people with ALS and their care partners were recruited through the ALS Society of British Columbia (ALS BC) communication channels (i.e., newsletters, mailing lists, social media). For this study, care partner was defined as a spouse or partner who provides live-in care to the person with ALS. Families (comprised of the person with ALS and their care partner) were included in the study if they were fluent in English, available for two 90-min assessments before and after a six-month period, and able to travel to the offices or mobile clinics of ALS BC to complete the assessments. Families were excluded from the study if the person with ALS was diagnosed with cognitive impairment or was in the pre-terminal or terminal stage of ALS. Families were also excluded if the person with ALS used a BiPAP machine for more than 2 h per day as it can be an indicator of respiratory decline and a shorter life expectancy for ALS patients [16]. Two families (four participants) did not qualify and were excluded from the study.

Families were assigned to either the control group (no respite care intervention) or the respite care group. An initial attempt was made to assign groups randomly; however, following reports that families were choosing not to participate because they did not wish to receive respite care, the decision was made to provide families with the option to choose after their baseline assessment whether they wanted to participate in the respite care intervention. Families choosing to participate in the respite care intervention received in-home respite care from a third-party service for a total of 6 months. For each month, 16 h of respite care were provided in which families could utilize and distribute however they liked. Families in the respite care intervention were able to customize a plan and choose from a wide range of healthcare and support services including but not limited to home-based rehabilitation, comprehensive nursing care, and day-to-day personal care and lifestyle support. Following the completion of the study, families in the control group were given the option to receive the respite care intervention if they liked. The final sample included 31 dyads (62 participants): 14 families in the control group and 17 families in the respite care group.

### Data collection

Semi-structured interviews and quantitative assessments were conducted at the offices and mobile clinics of ALS BC in British Columbia, Canada from August 2017 to April 2019. Face-to-face interviews and assessments were completely separately for the person with ALS and their care partner at two assessment points: baseline (prior to the provision of respite care) and after a six-month period.

#### Semi-structured interviews

Brief, semi-structured interviews were conducted with people with ALS and their care partners to explore the perspectives and lived experiences surrounding the impact of in-home respite care. An interview guide was developed by two members of the research team (JMR and EB) based on a review of the literature and in consultation with all team members and people with lived experiences of ALS. Open-ended questions were used to gain insight into the impact of respite care on the person with ALS and their care partner along the following themes: 1) challenges of ALS and caregiving, 2) perceptions of respite care, and 3) respite care experience. Interviews were conducted one-on-one, separately for the person with ALS and the care partner, with a trained research assistant and lasted approximately 15 min.

#### Quantitative measures

The Revised ALS Functional Rating Scale (ALSFRS-R) and Edinburgh Cognitive and Behavioural Screen (ECAS) were administered to measure the physical and cognitive functioning of people with ALS. Standardised validated assessments were used on people with ALS and their care partners to quantitatively assess the impact of respite care on a range of psychological measures including levels of anxiety, depression, quality of life, and care partner burden. All quantitative assessments and measures administered to participants are listed in Table 1.

**Table 1:** Quantitative assessments and measures

Administered to	Quantitative assessment	Measure(s)
People with ALS and Care Partners	GAD- 7 [17]	Worry and anxiety symptoms
	PHQ- 9 [18]	Major depressive symptoms
People with ALS	ALSFRS-R [19]	Functional impairment
	ECAS [20]	Cognitive and behavioural changes
	MQOL-R	Quality of life
	Personal Information Screen	Demographic information
Care Partners	Adapted SSLI	Support satisfaction and interactions
	ECAS – Section B [20]	Behavioural abnormalities in ALS patient
	Familial Information Screen	Demographic information
	QOLLTI-F v2 [21]	Quality of life
	Relationship Closeness Scale [22]	Closeness of patient-caregiver relationship
	ZBI [23]	Caregiver burden

*ALSFRS-R* Revised Amyotrophic Lateral Sclerosis Functional Rating Scale, *ECAS* Edinburgh Cognitive and Behavioural Amyotrophic Lateral Sclerosis Screen, *GAD-7* Generalized Anxiety Disorder 7-Item, *MQOL-R* McGill Quality of Life Questionnaire-Revised, *PHQ-9* Patient Health Questionnaire-9, *QOLLTI-F v2* Quality of Life in Life-Threatening Illness-Family Carer Version 2, *SSLI* Social Support List of Interactions, *ZBI* Zarit Burden Interview

### Data analysis

A total of 113 interviews over the two time points were conducted with people with ALS and their care partners. Eleven interviews were excluded from the analysis due to failure of audio recording, giving a total of 102 interviews for analysis. Our analysis was structured on a thematic analysis framework as proposed by Braun and Clarke [24]. Thematic analysis is a widely used approach that identifies patterns or themes within qualitative data [24]. In this study, we used thematic analysis to recognize, analyse, and report on frequent themes of the caregiving and respite care experience for people with ALS and their care partners. Following the framework, audio recordings of the interviews were first transcribed verbatim, and the first independent coder (JMW) familiarised themselves with the data. From this, a preliminary coding guide was developed by the first coder (JMW) based on 10% of the interviews (*n* = 10). The coding guide consisted of codes organized into a hierarchy of themes and subthemes. For each code, a definition was developed to assist with consistent coding. A second independent coder (MTT) applied the preliminary coding guide to the same set of interview transcripts to ensure reliability. Any discrepancies or disagreements were discussed and resolved through consensus by members of the research team (JMW, MTT and JMR). The coding guide was further refined through an iterative process to ensure all relevant themes and subthemes were captured. After an inter-rater reliability of at least 80% was achieved between the two coders (JMW and MTT), the remaining sample was split and independently coded by the two coders (JMW and MTT) using the final coding guide. The qualitative research software programme MAXQDA (VERBI GmbH, Berlin, Germany) was used to support the coding and qualitative analysis of the sample.

Descriptive statistical analyses were performed using Microsoft Excel to summarise participant characteristics and quantitative measures.

## Results

### Participant characteristics

Characteristics of people with ALS and their care partners are summarised in Table 2. At baseline, the mean age of people with ALS was 66 years (SD 9.3) and the majority identified as male (22/31, 71%) and white (24/31, 77%). On a scale of 0 (“worst”) to 48 (“best”), the mean score of physical functioning of daily activities (ALSFRS-R) was 31, a value associated with a 70 to 80% probability of nine-month survival [19]. On measures of cognitive and behavioural functioning, 45% (*n* = 14) of people with ALS showed abnormal performance (ECAS) with scores below abnormality cut-offs [20]. People with ALS showed 32% attrition between the first and second assessments. This was largely attributed to the death of people with ALS before the second assessment or choosing not to continue due to increased burden of research participation.

**Table 2:** Characteristics of people with ALS and their care partners at baseline

PEOPLE WITH ALS (*N* = 31)			
Sex			
Male	22	71.0%	
Female	9	29.0%	
Age (years)			
Mean (SD)	65.7 (9.3)		
Range	44–80		
Ethnic background			
White	24	77.4%	
Asian	4	12.9%	
Aboriginal	1	3.2%	
Black	1	3.2%	
Age of symptom onset (years)			
Mean (SD)	62.3 (9.5)		
Range	42–78		
Time from diagnosis to baseline (months)			
Mean (SD)	28.9 (33.6)		
Range	2–120		
Measures and Scales	Mean	St Dev	Range
ALSFRS-R	31.5	7.0	8–44
ECAS (total)	101.6	14.6	61–121
ECAS (ALS-specific)	80.3	12.1	46–97
ECAS (ALS non-specific)	21.3	4.3	13–30
CARE PARTNERS (*N* = 31)			
Sex			
Male	9	29.0%	
Female	22	71.0%	
Age (years)			
Mean	64.1 (SD 10.2)		
Range	42–81		
Ethnicity			
White	28	90.3%	
Asian	3	9.7%	
Currently employed			
Yes	7	22.6%	
No	24	77.4%	
Hours of care provided per week			
Mean	65.3		
Range	0–168		
Receiving caregiving assistance			
Formal (i.e., paid caregiver)	7	22.6%	
Informal (i.e., friends or extended family)	13	42.0%	

*ALSFRS-R* Revised Amyotrophic Lateral Sclerosis Functional Rating Scale, *ECAS* Edinburgh Cognitive and Behavioural Amyotrophic Lateral Sclerosis Screen

At baseline, the mean age of care partners was 64 years (SD 10.2) and the mean number of hours of care provided per week was 65.3 h**.** There was 13% attrition for care partners which was due to the death of their partner with ALS and their decision to withdraw from the study.

### Qualitative thematic analysis

**Fig. 1:**
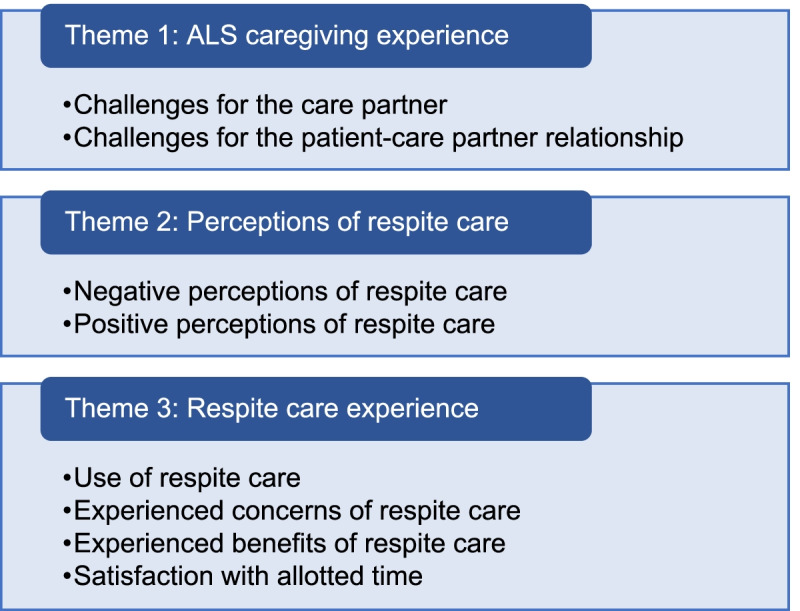
Themes and subthemes

Two main themes and constituent subthemes (Fig. 1) emerged from the analysis of the qualitative interviews. Findings are illustrated by verbatim quotes from the narratives and is denoted by participant ID (P, person with ALS; C, care partner), group (CG, control group; IG, intervention group) and interview time point (T1, baseline; T2, after 6 months). The data is represented as follows: the number of documents with the code (n) over the total number of relevant documents, percentage (%).

### Theme 1: ALS caregiving experience

All interviews with people with ALS and care partners at baseline and after a six-month period (*n* = 102) revealed insights about the ALS caregiving experience. Two subthemes were highlighted: challenges for the care partner and challenges for the patient-care partner relationship.

#### Challenges for the care partner

Several challenges of caregiving for the care partner were identified by people with ALS and care partners (77/102, 76%). Just over half the participants discussed caregiving challenges related to the psychosocial and emotional well-being of the care partner (55/102, 54%) including feelings of stress, helplessness, fear, sadness, and exhaustion. For many care partners, the emotional aspects of caregiving for a loved one was the most difficult component to cope with:

“The most difficult part for the me is the emotional part. It is hard to watch the woman that you married deteriorate and change right before your eyes. You have no control, and you can’t fix it. You can’t do anything about it. That I think is one of the hardest things that I have to deal with.” (C21, IG, T1)

“I don’t want to have a meltdown and end up a patient instead of caregiver. That’s where I’m scared that I’m headed.” (C19, IG, T1)

People with ALS and care partners also mentioned the need for time to be one of the greatest challenges for care partners (20/102, 20%). Committed to full-time caregiving, care partners struggled with not having enough time to pursue hobbies, meet other demands, or take a break:

“Getting time to myself. To do what I used to love and what I love to do. I don’t have time to do those things anymore [ … ] I kind of lost my sense of freedom that I really valued. I feel like I am always on call 24/7 and I can’t go anywhere.” (C11, IG, T1)

It was common for care partners to hold other life responsibilities such as work, volunteering, and taking care of family members in addition to caregiving. The ability for care partners to manage their lifestyle was an added challenge voiced by people with ALS and care partners (18/102, 18%):

“Managing my personal and work life. Being there for [partner] but also knowing I have to go work full-time, worrying about what is going on and when he is at home when I am at work, and the balance between the two.” (C8, CG, T1)

Care partners also struggled with the physical components of caregiving such as transferring their partner and battling physical exhaustion from limited sleep and rest (12/102, 12%). Some care partners even faced their own health problems (14/102, 14%) and failed to find the emotional capacity or time to attend to their own health needs:

“Last year, I had heart surgery and I ignored my own illness because I was more worried about this, and I almost died [ … ] it is just very difficult.” (C22, IG, T1)

#### Challenges for the patient-care partner relationship

Both people with ALS and their care partners discussed the challenges of ALS and caregiving for their relationship (41/102, 40%). Participants frequently emphasized the emotional challenges experienced in the relationship such as battling feelings of guilt, stress, and frustration towards their partner (33/102, 32%). One person with ALS explained how the shifting of household roles and routines in their relationship had created stress:

“With my partner now at home to help me, things were getting behind in the things that I would normally do because I can’t do them anymore [ … ] In turn, it would cause stress in our relationship.” (P14, IG, T2)

For the care partner, managing the roles as both a partner and caregiver often posed emotional difficulties in their relationship and sometimes the entire family:

“I have come to realize that my role is best suited not to be my wife’s primary caregiver. It has led to unhappiness on her part due to being highly critical, and it has led to mental health challenges for myself and my ability to be able to provide for my family [ … ] Basically, being a caregiver for my wife is killing me and it is destroying our family.” (C13, CG, T2)

People with ALS and care partners also discussed the challenge of increased dependency by the person with ALS (12/102, 12%). One person with ALS described how their dependency was changing the dynamic of their relationship:

“I am constantly with him and he is constantly at my beck and call. That doesn’t give him much independence at all. We have always been very close but being close and being demanding is a whole different thing.” (P15, IG, T1)

On the other hand, care partners often shared feelings of sympathy and guilt in response to their partner’s increased dependency:

“I find it very difficult to not come and go as a person does normally. To make any plans, I feel very badly for [him] and I feel selfishly myself that my life is just a standstill.” (C22, IG, T1)

### Theme 2: perceptions of respite care

Interviews at baseline for all people with ALS and care partners (*n* = 57) uncovered insights about the perceptions of respite care prior to the provision of service. Two subthemes were identified: negative perceptions of respite care and positive perceptions of respite care.

#### Negative perceptions of respite care

During the first interview at baseline, a third of people with ALS and care partners (21/57, 37%) raised barriers to the acceptance of respite care. The main barrier was privacy and comfort (10/57, 18%), with some participants expressing potential difficulty with having to adjust to a caregiver outside the family:

“I’m not too concerned about [respite care], it is just that I am a very private person and I have trouble asking other people besides my husband to do things for me.” (P15, IG, T1)

Some participants voiced that certain caregiving tasks such as bathing and dressing were more personal, and preferred the care partner to provide those forms of support:

“The only thing that she likes is she wouldn’t want them to bathe her. She wants me to do that. That’s about the only thing.” (C15, IG, T1)

For a few families who had previous experience with outside caregivers, a concern was the lack of consistency in care and staff members (8/57, 14%):

“I have used caregivers before, as I said, from both agencies and private help. What I find is, it is just a lot of times I have to explain things to people and it doesn’t really help me to relax in the beginning, especially when you transition through people.” (C33, IG, T1)

#### Positive perceptions of respite care

While barriers and concerns were discussed, the large majority of people with ALS and care partners (52/57, 91%) voiced the potential benefits of respite care. Participants expressed how they were willing to be flexible with their concerns and preferences to receive the expected benefits of the service:

“I am rather introverted, and I value my privacy. At the same time, I think I am willing to trade off a bit of that to get us some help once in a while.” (C29, CG, T1)

When asked, “In what way do you think respite care would be of most value to you and your partner?”, about half the participants (30/57, 53%) indicated that respite care could be helpful in providing a break for the care partner:

“[Respite care] will give my wife a big break because she has given up everything that she normally does to be with me and to care for me to make sure I am well. This will allow her to get a chance to go and do a few things that she rarely gets to do now.” (P11, IG, T1)

Other expected benefits of respite care included improved emotional well-being for the care partner (21/57, 37%), more independent time for the care partner (20/57, 35%), and improved emotional well-being for the person with ALS (13/36, 23%). One person with ALS described how they expected respite care to be a benefit for both themselves and their care partner:

“[Respite care] would probably make things easier on my wife. It would maybe make things easier on me down the road [ … ] I think doing that is going to make it easier for the people involved to live a good quality of life. Not only for me but for my wife.” (P8, CG, T1)

### Theme 3: respite care experience

Interviews after a six-month period with the respite care group (*n* = 24) revealed aspects of the respite care experience. Four subthemes were highlighted: use of respite care, experienced downsides of respite care, experienced benefits of respite care, and satisfaction with allotted time.

#### Use of respite care

Among the respite care group (*n* = 24), respite care was used to support families with basic and instrumental ADLs (17/24, 71%) including cleaning and maintaining the home, meal preparation, and managing the physical needs of the person with ALS. Respite care was also commonly used to provide time for the care partner to take a break (16/24, 67%) or attend to other life demands (8/24, 33%) such as work and family.

#### Experienced downsides of respite care

Nearly a third of people with ALS and their care partners (6/24, 29%) who received respite care discussed downsides to the service. The most common experienced concern was privacy and comfort (6/24, 25%) such as letting a non-family member into the home and having them perform caregiving tasks.

“You know when you are husband and wife, you trust each other. But, as a caregiver, somebody, a complete stranger, that is another story. You know, all of a sudden they are doing things with your body and there is that – well I would have the fear too.” (C24, IG, T2)

Lack of consistency in respite care staff was another experienced concern for some families in the respite care group (5/24, 21%). A few participants shared the challenge of having to work with several different staff members throughout the time of respite care. Having to repeatedly re-introduce a new person into the home was stressful, as well as inconvenient when staff members needed to be re-taught tasks and preferences.

“A downside was probably that even though you had three different people, they were all different. They were all good but readjusting and them re-learning what we wanted them to do in the house.” (P21, IG, T2)

A couple of families also faced scheduling issues that made their respite care experience more challenging (2/24, 8%):

“It was challenging with [the respite care] because of the fact that they cancelled several times because they didn’t have staff.” (C21, IG, T2)

#### Experienced benefits of respite care

Despite the challenges, people with ALS and care partners in the respite care group reported experienced benefits from the service (24/24, 100%). Nearly all participants (22/24, 92%) indicated that the provision of respite care benefited the patient-care partner relationship. In particular, both people with ALS and care partners expressed that the quality of their relationship improved due to respite care:

“I think that because of [respite care], [partner] and I are closer than we have been in years.” (P23, IG, T2)

The majority of participants (22/24, 92%) from the respite care group also reported benefits of respite care specific to the care partner. The most discussed benefit for the care partner was increased time for them to take a break, experience independence, and focus on other demands (22/24, 92%):

“I felt totally free - I could quickly go to shop something or go out of the house for a quick walk. I could just go to the bedroom, shut the door and do my own thing.” (C11, IG, T2)

Other participants shared how respite care had improved the emotional well-being of the care partner (14/24, 58%):

“It made me feel good to come home because it was like coming from a holiday and you’re feeling refreshed because I didn’t have to do what I normally would have been doing. I am happy. It was a treat. It was an absolute treat.” (C1, IG, T2)

Respite care was also discussed as a benefit for the person with ALS (13/24, 54%). Some participants articulated that respite care helped provide companionship to the person with ALS, making the person with ALS feel more connected (8/24, 33%):

“It is good for the patient too. It is a little bit of a social thing and it is good to have another person around to just come and chat.” (P21, IG, T2)

People with ALS and care partners also commented on how respite care improved the emotional well-being of people with ALS (5/24, 21%):

“A reduction of stress. It was a big thing knowing that we have someone supporting us in the areas that we were lacking. It was certainly comforting. It is the consistency of knowing that things are being done. It alleviates that stress.” (P14, IG, T2)

#### Satisfaction with allotted time

In response to the question “Did you feel like 16 hours a month was enough time for respite care?”, the majority of people with ALS and care partners (15/24, 63%) from the respite care group stated they were satisfied with the amount of time provided; however, among these participants, half of them (8/24, 33%) anticipated more time being needed in the future as the disease and caregiving demands progress:

“For where his illness was at the moment, four hours a week was helpful. I can see down the road, when his illness is going to get worse – not if, but when – that we would feel that we need more than that.” (C14, IG, T2)

A third of people with ALS and care partners (8/24, 33%) expressed that 16 h a month of respite care was not enough time to meet their caregiving needs. Many pointed to the rapid progression of ALS in their partner as a reason for needing more support:

“In the beginning, it was okay but as he deteriorated, it wasn’t enough.” (C20, IG, T2)

### Quantitative analysis

Due to challenges with recruitment, the quantitative data of the study was not powered to detect differences within or between the control group and respite care group. The full set of descriptive results is shown in Table 3. While we cannot interpret for significance, the following trends were observed between the two time points: care partners in the control group reported a 10.6% increase in their anxiety symptoms while care partners in the respite care group reported a 10.8% decrease in their anxiety symptoms (Generalized Anxiety Disorder 7-Item), care partners in the control group reported a 5.4% increase in their major depressive symptoms while care partners in the respite care group reported a 4.7% decrease in their major depressive symptoms (Patient Health Questionnaire-9), and people with ALS in the control group reported a 17.3% increase in functional impairment while people with ALS in the respite care group reported a 9.5% increase in functional impairment (ALSFRS-R).

**Table 3:** Descriptive statistics for quantitative assessments at baseline and after six months

CONTROL GROUP								
**People with ALS (*N*** **= 14)**								
	Time 1 (baseline)				Time 2 (after six months)			
Assessment	N	Mean	St Dev	Range	N	Mean	St Dev	Range
ALSFRS-R	13	34.2	6.6	23–44	8	26.1	8.5	12–37
ECAS	12	104.1	12.6	82–120	5	114.4	7.5	108–127
GAD-7	13	3.3	5.1	0–15	8	2.3	4.1	0–11
MQOL-R	13	6.2	0.7	5.2–7.8	8	5.8	0.6	4.6–6.6
PHQ-9	13	6.5	3.9	0–12	8	5.4	4.3	1–15
**Care Partners (*N*** **= 14)**								
	Time 1 (baseline)				Time 2 (after six months)			
Assessment	N	Mean	St Dev	Range	N	Mean	St Dev	Range
Adapted SSLI	13	14.7	3.8	10–23	9	15.3	2.4	12–19
ECAS – Section B	13	1.4	1.5	0–5	9	1.1	1.5	0–4
GAD-7	12	6.6	5.3	0–18	10	6.5	4.4	1–15
PHQ-9	13	6.8	6.0	0–19	10	7.6	6.1	1–19
QOLLTI-F v2	13	5.6	1.1	3.9–7.6	10	6.0	0.5	4.7–6.6
Relationship Closeness Scale	13	16.0	2.2	13–19	10	16.5	1.8	13–19
ZBI	13	34.6	16.3	9–56	10	33.4	14.0	7–51
RESPITE CARE GROUP								
**People with ALS (*N*** **= 17)**								
	Time 1 (baseline)				Time 2 (after six months)			
Assessment	N	Mean	St Dev	Range	N	Mean	St Dev	Range
ALSFRS-R	17	29.5	6.9	8–37	12	26.0	9.5	11–41
ECAS	16	99.2	16.4	61–121	8	104.6	12.2	79–118
GAD-7	17	5.1	5.8	0–19	12	3.3	2.9	0–9
MQOL-R	17	6.4	1.3	4.5–9.1	12	6.5	0.8	5.2–7.7
PHQ-9	17	6.5	6.4	0–20	12	5.3	4.1	0–14
**Care Partners (*N*** **= 17)**								
Assessment	Time 1 (baseline)				Time 2 (after 6 months)			
	N	Mean	St Dev	Range	N	Mean	St Dev	Range
Adapted SSLI	17	14.5	4.1	9–24	16	15.5	5.2	6–24
ECAS – Section B	17	1.2	1.1	0–4	16	1.1	1.0	0–3
GAD-7	17	8.7	5.7	0–21	16	6.6	4.2	0–15
PHQ-9	17	10.2	5.9	11–24	16	8.9	5.8	2–21
QOLLTI-F v2	17	5.8	0.8	4.5–7.0	15	5.8	0.7	4.6–5.8
Relationship Closeness Scale	17	16.4	2.0	13–20	16	17.1	2.1	14–20
ZBI	17	41.5	13.4	23–70	16	38.6	12.3	24–60

*ALSFRS-R* Revised Amyotrophic Lateral Sclerosis Functional Rating Scale, *ECAS* Edinburgh Cognitive and Behavioural Amyotrophic Lateral Sclerosis Screen, *GAD-7* Generalized Anxiety Disorder 7-Item, *MQOL-R* McGill Quality of Life Questionnaire-Revised, *PHQ-9* Patient Health Questionnaire-9, *QOLLTI-F v2* Quality of Life in Life-Threatening Illness-Family Carer Version 2, *SSLI* Social Support List of Interactions, *ZBI* Zarit Burden Interview

## Discussion

Findings from the qualitative analysis of this study emphasize (1) the unique caregiving challenges for people with ALS and their care partners, (2) the experienced benefits and concerns surrounding respite care, and (3) the need to prioritize the design, delivery, and evaluation of respite care with the ALS community.

Narratives of people with ALS and their care partners revealed specific caregiving challenges for the care partner and the patient-care partner relationship. Consistent with other research, care partners expressed significant challenges related to their emotional well-being and need for more personal time [1, 25]. Many care partners found it difficult to manage full-time caregiving with other life responsibilities and sacrificed personal hobbies and commitments to meet the increasing demands of caregiving. Particularly salient was the deep connection and sense of responsibility care partners held as both a spouse and caregiver. With care partners taking on this dual role, caregiving challenges were often emotional in nature and directly impacted the patient-care partner relationship. For people with ALS, a deep concern was the fear and guilt of becoming more dependent on their care partner as their illness rapidly progressed. These findings are supported by the literature that have found the emotional well-being of people with ALS to be tied to the perceived well-being of their partner [25, 26]. In a study by Ando et al. (2018), people with ALS reported they had greater concern for their significant other over themselves, and the perceived well-being of their partner was an important contributor to their quality of life and psychological well-being. Taken together, our results further illustrate that caregiving challenges in ALS do not just greatly impact the care partner but extend to people with ALS and the patient-care partner relationship.

The potential for respite care to mitigate caregiving challenges and support people with ALS and their care partners was highlighted in this study. Prior to the provision of respite care, most participants expressed how respite care could be of positive value to themselves and their partner. Aligned with these expectations, all people with ALS and their care partners receiving the respite care intervention reported experienced benefits from the service including improved relationship quality, more time for the care partner to pursue personal commitments or take a break, and improved well-being for the person with ALS and the care partner. In combination with results from other studies of respite care [11, 27], our findings showcase respite care as a critical tool to mitigate the burden and support the needs of families with ALS.

Despite the experienced benefits, some barriers and downsides were discussed by participants. The lack of privacy from letting an outside caregiver into the home made some families feel hesitant about the service. Moreover, some families voiced a lack of consistency in respite care staff and challenges with scheduling. These findings are aligned with other studies that have found privacy and staff reliability to be key barriers to seeking professional care among ALS care partners [12, 25, 27]. Taken together, our findings in combination with other research emphasize the need to address these barriers in program planning and implementation. Health care professionals and policy makers play an integral role in the access, quality, and credibility of respite care services [28]. It is critical for institutional decision-makers and program staff to prioritize the trust and unique care needs of families with ALS. This can be supported by maintaining consistency in staff members, upholding clear communication between staff and care partners, and building a collaborative care partnership that acknowledges the centrality of families and their care preferences [29, 30]. Prioritizing engagement with the ALS community in the design, delivery, and evaluation may help respite care better meet the needs of families and improve service uptake.

Overall, the present findings highlight that the experienced benefits of respite care far outweighed the risks. Although people with ALS and care partners expressed downsides to the service, many voiced that the benefits of respite care were of greater value than their concerns. Our study also uncovered an increasing need for respite care services among the ALS community. Over half of the participants in the respite care intervention indicated they needed more time for respite care than what was allotted, or they anticipated more time being needed in the future. Given the rapid progression of ALS and the increasing need for support, it is essential families are well-informed of the potential benefits and risks of respite care early in their care and decision-making. Future work should focus on the evaluation of these services to improve efforts in funding, availability, and program quality.

### Limitations

Due to a small pool of participants to recruit from, we did not exclude families who had previous experience using respite care. As a result, we cannot tease out if participants’ described experiences with respite care were those from the study’s intervention specifically or from other past experiences of respite care. We can, however, confirm that all participants in the control group had never received respite care prior to their participation in the study. In addition, the ability for families to choose whether they wanted to receive the respite care intervention may have created a positive bias towards our findings on the respite care experience. We would also like to acknowledge the lack of ethnic diversity in our participants. Future work should explore the perceptions and impact of respite care on families from a wider range of cultural backgrounds. Finally, the quantitative component of this study was underpowered, and we support further investigation to quantitatively assess the impact of respite care on people with ALS and their care partners.

## Conclusion

ALS brings upon unique caregiving challenges for people with ALS, their care partners, and the patient-care partner relationship. The present study highlights respite care as a critical tool to alleviate these challenges and support the needs of families with ALS. Overall, our findings reveal benefits including improved quality of the patient-care partner relationship, increased personal time for the care partner, and improved well-being for both the care partner and person with ALS. However, we also uncovered important barriers and concerns surrounding the service such as lack of privacy and staff consistency. Health care professionals and policy makers play an important role in addressing these barriers to improve respite care uptake, funding, and accessibility. In pursuit of greater patient- and family-centred care, it is important for the ALS community to be actively engaged in the design, delivery, and evaluation of these services. Future work should investigate best practices for rigorous evaluation and aim to quantitatively assess the impact of respite care for people with ALS and their care partners.

## Data Availability

The datasets generated and/or analysed during the current study are not publicly available because the data are confidential, but are available from the corresponding author on reasonable request.

## Abbreviations

ALS: Amyotrophic lateral sclerosis
ALSFRS-R: Revised Amyotrophic Lateral Sclerosis Functional Rating Scale
ALS BC: ALS Society of British Columbia
ECAS: Edinburgh Cognitive and Behavioural Screen
